# From trauma to pain - a pathway to dental anxiety

**DOI:** 10.1192/j.eurpsy.2021.492

**Published:** 2021-08-13

**Authors:** A.T. Pereira, C. Cabaços, A. Araujo, M.J. Soares, A. Macedo

**Affiliations:** 1 Institute Of Psychological Medicine, Faculty Of Medicine, University of Coimbra, coimbra, Portugal; 2 Psychiatry Department, Centro Hospitalar e Universitário de Coimbra, Coimbra, Portugal

**Keywords:** Dental Anxiety, trauma

## Abstract

**Introduction:**

The main risk factor for dental anxiety is previous traumatic experiences of pain in the dental office. Other consistent etiologic factors are trait-anxiety and preparedness (genetic predisposition to increased sensitivity to pain and aversive stimuli). However, there is a wide inter-individual diversity in the anxiety experience – not all individuals with traumatic experiences at the dentist will develop dental anxiety anxiety

**Objectives:**

To explore potential paths by which a traumatic experience at the dentist (TRAUMA) can lead to dental anxiety.

**Methods:**

A community sample of 552 adults (68.2% women; mean age= 35.15±15.790) completed the Portuguese validated versions of: Dental Fear Survey/DFS, State-Trait Anxiety Inventory, Sensitivity to Pain Traumatization/SPT Scale, Fear of Dental Pain/FDP Questionnaire and Perseverative Thinking Questionnaire–15.

**Results:**

140 participants (25.2%) had TRAUMA; it was significantly (p<.01) correlated with trait-anxiety (Spearman r=.190), SPT (r=.192), FDP (r=.333), RNT (r=.274) and dental anxiety (DFA total score; r=.418). In the mediation analysis (PROCESS macro 3.5 for SPSS; Model 81; Hays, 2020), trait-anxiety and gender were controlled (as RNT, SPT, FDP mean scores were higher in women, p>.01). Our model was significant (R^2^=17.15%; p<.001) and showed that TRAUMA predicted dental anxiety directely [direct effect: 10.25 (95% CI - 7.10-13.40)] and also through SPT, FDP and RNT (5 significant indirect effects).

**Conclusions:**

This study underlines the importance of avoiding traumatic experiences in the dental office and of good clinical communication in pain management. If trauma still occurs, dentist should learn how to reduce its impact on the sensitivity and fear of pain and on the RNT.

